# Effects of Lean Thinking and Emerging Technologies on Breast Cancer Patients' Therapeutic Process During COVID-19 Pandemic: A Case-Control Matched Study

**DOI:** 10.3389/fsurg.2021.582980

**Published:** 2021-03-11

**Authors:** Francesca Pellini, Giacomo Di Filippo, Sara Mirandola, Giulia Deguidi, Elisa Filippi, Giovanni Paolo Pollini

**Affiliations:** Complex Operative Unit of Breast Surgery, Breast Unit, Oncologic Surgery Department, AOUI, Ospedale Civile Maggiore, Verona, Italy

**Keywords:** breast cancer, COVID-19, lean management, indocyanin green, coronavirus (2019-nCoV), pectoral nerve block

## Abstract

**Introduction:** The advent of the COVID-19 pandemic has led to the sudden disruption of routine medical care, and the subsequent reorganization of hospital structures and therapeutic algorithms, aiming at protecting patients and health professionals. This was inevitably bound to affect our Breast Unit, dilating both pre- and post-operative times. The aim of this study was to evaluate the effect on patients' flow of organizational and logistic changes (*key interventions*) based on lean thinking implemented after the COVID-19 outbreak.

**Materials and Methods:** Clinical and demographic data were retrospectively collected from patients undergoing sentinel lymph node biopsy for breast cancer at the Verona University Hospital from January 2018 to June 2020. Patients enrolled (*n* = 341) were divided into two groups according to date of admission: before (Group A; *n* = 294) and after (Group B; *n* = 47) the implementation of key interventions. Each case in Group B was subsequently matched 1:1 by means of case-control matching with cases from Group A for age, comorbidities, and type of surgery (Subgroup A1; *N* = 47). Pre-admission time (T0) and length of stay (T1) were compared between the three groups.

**Results:** Median T0 was 312 h, whereas median T1 was 24 h. Patients in Group B had a higher frequency of comorbidities (57.4 vs. 25.2%, *p* = 0.001) and underwent mastectomy more often than patients in Group A (61.7 vs. 36.7%, *p* = 0.001). Both median T0 and T1 were higher in group B than in group A (384 vs. 300 h, *p* = 0.001, 48 vs. 24 h, *p* = 0.001, respectively). Median T0 and T1 did not significantly differ between Group B and Subgroup A1 (all *p* > 0.05).

**Conclusions:** Lean thinking and new technologies could prove useful to the optimization of preoperative and postoperative times during the current pandemic, minimizing healthcare personnel and patients' exposure to SARS-CoV-2, and promoting a rational use of limited resources, while complying with oncological principles.

## Introduction

Breast cancer affects a large number of women worldwide. In Italy in particular it is by far the most common cancer, with 53,500 cases reported in 2019 alone (1). The growing trend toward the centralization of breast cancer care in multidisciplinary breast units has benefited treatment outcomes, likely due to a greater adherence to practice guidelines. However, the patterns of care that are actually provided in said multidisciplinary breast units and their associated costs have seldom been evaluated (2). The achievement of the desired health outcomes depends on multiple factors, including the availability of resources, the health service organizational structure, and the type of hospital admission (day surgery, week surgery, ordinary hospitalization or day services), which in turn are influenced by the complexities of healthcare management.

A feasible way to reduce waste and inefficiency in breast cancer management is the adoption of lean methodologies, such as total quality management, lean thinking, six sigma, and process management with a reengineering or improvement approach. These organizational philosophies share the concept of “quality” (3), which consists in acknowledging patients' needs and identifying and containing medical errors, while still retaining a global vision of the processes. Lean thinking refers to a management philosophy aimed at eliminating redundant and unnecessary activities. Lean management allows for better performance, increasing both the efficiency and quality of services, while reducing bottlenecks, imperfections, and lead times. This system relies on two pillars: the creation of value and the elimination of waste. The lean system encourages operators to question company performance and management, aiming at the continuous improvement of operational processes and customers' satisfaction through the provision of quality services at a lower cost, while eliminating waste. Criticalities in the management of breast cancer surgery candidates can be identified by using a process flow map (4). Prior research has shown how the use of lean thinking in breast cancer surgery is not common, especially in Italy.

Six Sigma is a popular quality improvement methodology developed in the mid-1980s, whose aim is the reduction of errors. Six Sigma measures quality in terms of defect rates and sets a target error rate of no more than 3.4 defects per million opportunities or 6 standard deviations from the process mean. Six Sigma relies on statistical rigor to determine sources of variation: data is collected and analyzed multiple times after each process modification to identify improvements in error rates.

Lean thinking and Six Sigma are complementary concepts that can be combined to create Lean Six Sigma, a methodology that benefits from the statistical rigor of Six Sigma as well as the cyclical waste reduction of the Lean methodology (3, 5).

The unexpected advent of the coronavirus disease-2019 (COVID-19) pandemic has led to a sudden disruption of routine medical care, with a subsequent reorganization of hospital structures and resettlement of therapeutic algorithms. Extraordinary measures have been advocated to protect patients and health professionals, and to create a safe path to treat patients.

The current healthcare emergency has also become an opportunity for the application of new technologies (e.g., indocyanine green and cryoablation) in clinical practice to improve the patient's management flow, despite the limited resources.

The aim of the present study is to compare, through performance measurements, breast cancer patients' workflow times at our Breast Unit prior to the outbreak of the COVID-19 pandemic with the workflow times during the COVID-19 pandemic after the implementation of some key interventions.

## Materials and Methods

For the purposes of this study, a multidisciplinary working group was created, which included a project manager, a director of breast surgery, a breast care nurse, and a medical director. The team conducted a study on lead times of patients admitted to the Breast Surgery Unit, Surgical Oncological Department in Verona, Italy, before and during the COVID-19 pandemic.

An analysis of patients' flow processes for breast surgery was performed to uncover critical issues and propose possible improvements. The main objective of the study was to identify and monitor improvements in the care processes achieved through organizational and logistic changes (*key interventions*) based on lean thinking (Table 1). These key interventions have been implemented since March 16, 2020.

**Table 1:** Key interventions implemented and their possible impact on flow times.

**Key intervention**	**Preoperative**	**In-hospital stay**	**Postoperative**
Multidisciplinary discussion for priority assessment	+		
Routine PNB during surgery		+	
Routine use of ICG fluorescence guided sentinel lymph node biopsy	+		
Interruption of group postoperative physiotherapy sessions, replaced by physiatric evaluation only			+
Favoring phone consultation over outpatient visits	+/−		+/−

*PNB, ultrasound guided pectoral nerve block; ICG, indocyanine green*.

**Figure 1:**
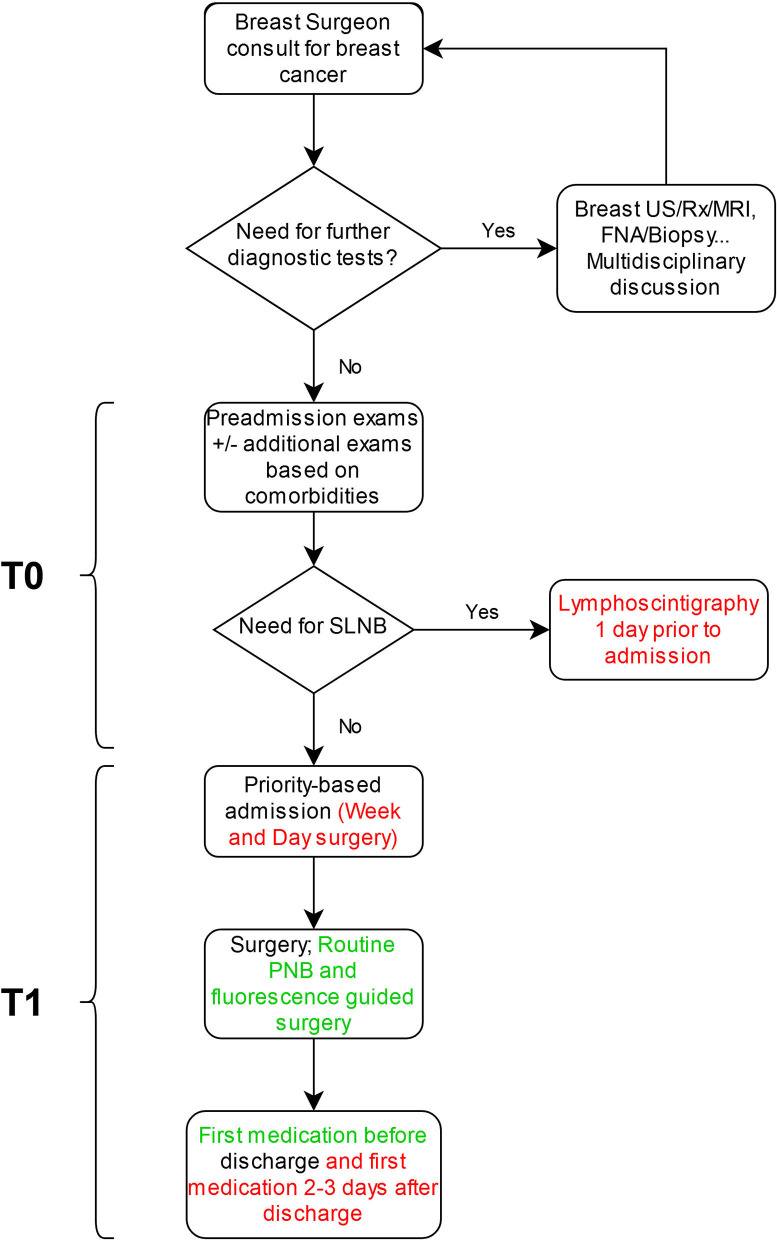
Breast cancer patients' work-flow. Red: Elements removed from practice during the COVID-19 pandemic; Green: Elements added to practice during the COVID-19 pandemic. SLNB, sentinel lymph node biopsy; PNB, pectoral nerve block; US, ultrasound; Rx, mammography; MRI, magnetic resonance imaging, FNA, fine needle aspiration.

Our Unit workflow before and after the implementation of key interventions is illustrated in Figure 1.

Patients' anamnestic and clinical information (age, sex, comorbidities, type of surgery and date of admission, surgery, and discharge) were collected from paper or electronic medical records and the prospectively filled digital information system database (dataBreast) of our Unit.

From September 2018 to June 2020, 613 consecutive patients underwent surgery for breast cancer at our Unit. Among them, 414 patients underwent sentinel lymph node biopsy (SLNB). Exclusion criteria were: age <18 years, SLNB as part of a second surgical operation, incomplete data. Our study population included 341 patients. We divided the population into 2 groups according to date of admission: before the implementation of key interventions (Group A; *N* = 294) and after the implementation of key interventions (Group B; *N* = 47). To avoid possible biases, every case in Group B was matched 1:1 by means of case-control matching with 47 cases from Group A for age (±5 years), comorbidities, and type of surgery (Subgroup A1; *N* = 47). We then compared the time periods involved in the diagnostic and therapeutic processes of the three groups. T0 (hours) was defined as the time between the clinical evaluation in which the surgical indication was given and the date of admission. T1 (hours) was defined as the time between surgery and discharge. Comorbidities were defined as any concurring medical condition affecting the patient.

Categorical data was expressed as frequency and relative percentage. Continuous data was reported as median and interquartile range. Demographic and clinical data were compared between groups with χ^2^ test for categorical variables and Mann Whitney test for continuous variables. A linear regression analysis was performed to identify predictors of T0 and T1 in both the unmatched and matched cohorts.

Data was analyzed using SPSS v. 25 (IBM Corp. Released 2017. IBM SPSS Statistics for Windows, Version 25.0. Armonk, NY: IBM Corp.).

Written informed consent was obtained from all patients.

## Results

During the COVID-19 pandemic, our surgical activity was reduced by 8.1%. The medical and surgical activity was not relocated to any other institution.

Our study population included 341 patients with a median age of 60 years (50–73). The majority of patients underwent conservative treatment for breast cancer (59.8%) and did not have any significant comorbidity (70.4%). Median T0 was 312 h (192–456), whereas median T1 was 24 h (24–48).

Patients were divided into two groups according to time of admission (Table 2). Patients in Group B had more comorbidities (57.4 vs. 25.2%, *p* = 0.001) and underwent mastectomy more frequently than patients in Group A (61.7 vs. 36.7%, *p* = 0.001). Both median T0 and T1 were higher in Group B than in Group A (384 vs. 300 h, *p* = 0.001 and 48 h vs. 24 h, *p* = 0.001, respectively) (Table 2).

**Table 2:** Unmatched populations' characteristics.

		**Key interventions**		
		**Group A (*N* = 294)**	**Group B (*N* = 47)**	
		**Median (IQR), N (%)**	**Median (IQR), N (%)**	***p***
Age, years		59 (49–72)	62 (50–77)	0.28
Comorbidities	No	220 (74.8)	20 (42.6)	0.001
	Yes	74 (25.2)	27 (57.4)	
Type of surgery	BCS	186 (63.3)	18 (38.3)	0.001
	Mastectomy	108 (36.7)	29 (61.7)	
T0, hours		300 (192–432)	384 (288–504)	0.001
T1, hours		24 (24–48)	48 (24–72)	0.002

*IQR, interquartile range; BCS, breast conserving surgery*.

Patients in Group B were matched 1:1 with patients in Group A (Subgroup A1) to account for possible biases, as previously described (Table 3). Both median T0 and T1 did not significantly differ between Group B and Subgroup A1 (*p* > 0.05).

**Table 3:** Matched populations' characteristics.

		**Key interventions**		
		**Subgroup A1 (*N* = 47)**	**Group B (*N* = 47)**	
		**Median (IQR), N (%)**	**Median (IQR), N (%)**	***p***
Age, years		62 (49−75)	62 (50–77)	0.90
Comorbidities	No	20 (42.6)	20 (42.6)	1.00
	Yes	27 (57.4)	27 (57.4)	
Type of surgery	BCS	18 (38.3)	18 (38.3)	1.00
	Mastectomy	29 (61.7)	29 (61.7)	
T0, hours		360 (192–528)	384 (288–504)	0.42
T1, hours		48 (24–72)	48 (24–72)	0.37

*IQR, interquartile range, BCS, breast conserving surgery*.

A univariate linear regression analysis showed type of surgery to be the sole predictor of T1 both in Group B and in Subgroup A1, whereas no predictors were found to be associated with T0 in Group B nor in Subgroup A1 (Table 4). A subgroup analysis of T0 and T1 differences before and after the implementations of key interventions did not show any statistically significant difference within the analyzed parameters (Table 5).

**Table 4:** Univariate linear regression analysis for T0 and T1 within subgroup A1 and group B.

		**Subgroup A1**			**Group B**		
		**B**	**CI 95%**	***p***	**B**	**CI 95%**	***p***
T0	Type of surgery	60.05	(−71.32 to 191.41)	0.36	23.86	(−114.29 to 162.02)	0.73
	Age	2.48	(−1.84 to 6.79)	0.25	1.25	(−3.27 to 5.77)	0.58
	Comorbidities	84.22	(−43.67 to 212.11)	0.19	50.00	(−85.19 to 185.19)	0.46
T1	Type of surgery	35.91	(29.03–42.79)	0.0001	35.86	(22.51 to 49.22)	0.0001
	Age	−0.25	(−0.67 to 0.17)	0.23	0.02	(−0.54 to 0.58)	0.94
	Comorbidities	1.2	(−11.37 to 13.77)	0.85	2.31	(−14.54 to 19.16)	0.78

*CI, confidence interval*.

**Table 5:** Differences in T0 and T1 among subgroup A1 and group B.

		**T0**			**T1**		
		**Subgroup A1**	**Group B**		**Subgroup A1**	**Group B**	
		**Median (IQR)**	**Median (IQR)**	***p***	**Median (IQR)**	**Median (IQR)**	***p***
Age	<62	336 (144–456)	360 (216–480)	0.33	48 (24–72)	48 (24–72)	0.78
	≥62	444 (276–588)	384 (300–504)	0.82	48 (24–72)	48 (24–72)	0.32
Type of surgery	BCS	336 (168–528)	360 (216–456)	0.35	24 (24–24)	24 (24–24)	0.55
	Mastectomy	360 (264–480)	384 (312–504)	0.87	72 (48–72)	72 (48–72)	0.54
Comorbidities	No	336 (144–444)	360 (336–444)	0.56	48 (24–72)	48 (24–72)	0.58
	Yes	360 (240–648)	384 (216–504)	0.51	48 (24–72)	48 (24–72)	0.38

*IQR, interquartile range; BCS, breast conserving surgery*.

## Discussion

Lean thinking is a management strategy applicable to any organization as a way of optimizing every aspect of the patient's process flow, allowing for continuous improvements aimed at creating value for the patient (6).

The COVID-19 pandemic has prompted a sudden change in the organization of healthcare facilities and the management of patients in order to limit the spread of the virus.

The Italian healthcare system's efforts have been directed toward assisting SARS-CoV-2 infected patients while guaranteeing adequate treatment to non-deferrable oncologic and emergency cases. During the current pandemic situation, it has been of paramount importance to redesign patient flow and review priorities, while respecting quality standards and guaranteeing safety and efficiency. Different organizations and societies have promptly produced international guidelines on the management of elective surgeries (7–9), trying to define a clear pathway to follow in this extremely difficult and unusual situation. All these different guidelines shared the common goal of striking a balance between minimizing patient's and physician's exposure to SARS-CoV-2 and promoting a rational use of hospital resources.

In order to protect patients and healthcare professionals, and to redirect all resources (human, spatial, and economic) to the care of COVID-19 patients, our hospital has decided to suspend day and week surgery admissions, relocate some specialists from their original wards to *ad hoc* newly created COVID-19 wards, and limit patients' access to the hospital to avoid gatherings and respect social distancing. This was inevitably bound to affect our routine as a Breast Unit, dilating both pre- and post-operative times.

Likewise, other Breast Units made several adjustments to their clinical practice in order to limit patients and staff members' risk of SARS-CoV-2 infection. Such adjustments included preadmission phone screenings, dedicated in-hospital pathways, and social distancing measures. Admission times have been shortened by implementing day surgery regimens for eligible patients and by entrusting wound care to general practitioners (10).

Our Breast Unit decided to face the advent of the COVID-19 pandemic and subsequent hospital organizational changes by conceiving and implementing some key interventions. The key interventions implemented have been collegially discussed and are widely evidence-based.

First and foremost, we thought that a multidisciplinary discussion was needed to define priorities among breast cancer treatments, thus providing patients with the best possible health outcomes. This has been a concern shared by other surgical specialties as well, highlighting the common will to enforce social distancing and preserve hospital resources without jeopardizing patients' health (11). Therefore, identifying which breast cancer patients required more urgent care and which could wait until the pandemic was over was of pivotal importance. Although international guidelines (12, 13) for the triage of breast cancer patients had provided useful indications on the management of breast cancer surgery candidates, they had to be adapted to the single specific setting, considering availability of hospital resources and local impact of the COVID-19 pandemic.

Provided that surgery is still the cornerstone of breast cancer management, it should continue to follow standard protocols, as long as they do not conflict with the new institutional policies drafted for the concurrent pandemic crisis.

In order to better guarantee both patient and physician's safety during the COVID-19 pandemic, we followed the triage guidelines proposed by the European School of Oncology (12).

After discussion by a multidisciplinary team, patients completing neoadjuvant treatment, clinical stage T2 or N1 ER+/PR+/HER2 negative tumors, triple-negative or HER2 positive patients, discordant biopsies likely to be malignant, and patients with locoregional recurrence were prioritized over excisions of benign lesions, fibroadenomas, papillomas, high risk lesions with atypia, and prophylactic surgeries (risk reducing surgeries). According to international guidelines and current literature, patients with luminal-like breast cancer (HER2 negative, ER+) could benefit from neoadjuvant endocrine therapy (14) as a safe and effective option that would allow them to temporarily delay surgery and outpatient visits. Additionally, current literature suggests that tamoxifene may not be associated with a higher rate of endometrial cancer in women with breast cancer compared with women treated with aromatase inhibitors or who received no treatment (15). This breast cancer management strategy was applied to the current peculiar pandemic situation at our Unit, allowing us to overcome priority concerns in selected patients by deferring surgery without jeopardizing oncological outcomes.

In a recent editorial, Holmes (16) suggested that, during the current pandemic, cryoablation therapy could either be a definitive solution or a valid stopgap solution in patients too anxious to delay surgery. Cryoablation has progressively emerged as a minimally invasive new technology alternative to breast cancer surgery, with reduced morbidity and psychosocial and cosmetic impact. There is compelling evidence (17, 18) that cryoablation might represent a valid alternative to surgical resection for small luminal-like tumors as a definitive treatment. This procedure has the potential for minimizing the risk of disease progression of the primary tumor site, while reducing the anxiety of a prolonged surgical delay and saving healthcare resources. Although cryoablation therapy was not the main focus of the present study, our Unit has endorsed this procedure as a stopgap solution allowing for the delay of surgical treatment while reducing morbidity, costs, and patient and healthcare personnel's exposure to SARS-CoV-2. We believe this emerging technology might also represent a valid tool in the management of breast cancer when dealing with limited resources, such as during the current pandemic.

Breast cancer diagnosis is an extremely stressful event. These patients represent a vulnerable population who could be psychologically affected by the measures implemented to manage the COVID-19 pandemic. The impact on mental health of the delay of surgical treatment these measures entail should not be underestimated. As current literature suggests, patient management should include mental health as well, and potential psychological consequences should be considered before implementing said measures (19). In this regard, we believe that the aforementioned solutions (i.e., cryoablation and neoadjuvant endocrine therapy) could represent a valuable asset to address psychological concerns during the current pandemic in addition to proper mental health care by a dedicated professional team.

Due to its evidence-based sensitivity, specificity, and cost-effectiveness (20–22), the use of Indocyanine Green fluorescence guided surgery (ICG) has been demonstrated to be a valid alternative to lymphoscintigraphy, which was routinely used at our Unit for sentinel lymph node detection. The fact that this procedure can be done directly in the operating room, thus not requiring patient's pre-hospitalization, explains why we thought that its preferential use over lymphoscintigraphy could significantly impact preoperative times. Additionally, by avoiding lymphoscintigraphy, our aim was to benefit both the patients (saving them a trip to the hospital) and the healthcare personnel (decreasing patient-patient and patient-physician interaction).

Among the other key interventions, the choice to execute ultrasound guided pectoral nerve blocks (PNB) during mastectomies was based on the ever-growing body of literature that, over the years, has demonstrated how PNB is associated with a greater postoperative pain control (23–26), thus improving patients' perceived quality of the assistance received and possibly reducing postoperative stay.

Data presented in this study show that the key interventions implemented managed not to lengthen hospitalization times both before and after surgery.

It must be highlighted that patients who were treated after the implementation of the key interventions had a significantly higher rate of comorbidities and underwent mastectomies more often than patients before the implementation of those measures. This might be due to many concurring factors, such as the strong need to prioritize patients with the highest chances of worse oncological outcomes. Another possible explanation is that, due to the COVID-19 pandemic, when faced with the choice between breast conserving surgery and subsequent radiotherapy or mastectomy and immediate reconstruction, most patients chose to solve their problem in a single session, without having to go back to the hospital for radiotherapy. Both comorbidities and type of surgery have a strong impact on pre-admission times and length of hospital stay, hence the need for case-control matching.

To the best of our knowledge, this is the first case-control matched study to analyze the effects of lean thinking and the application of emerging technologies on breast cancer patients' diagnostic and therapeutic process during the current pandemic, and to provide insights on their strengths. However, this study is subject to a few limitations that should be overcome in future research. The primary limitation of our study is the fact that we lack a control cohort of patients managed after the implementation of institutional anti SARS-CoV-2 measures but before the implementation of the key interventions. This is due to the fact that the implementation of the key interventions and of institutional measures nearly coincided. Additionally, although somewhat urged by the current pandemic situation, the simultaneous introduction of many key interventions could possibly adversely affect the monitoring process lean thinking is based upon, by inadvertently disregarding potential confounders and their effects on multiple steps of the investigated process.

## Conclusions

During the current COVID-19 pandemic, we applied lean thinking to the management of breast cancer surgery candidates at our Center. We provided an example of how this strategy could be implemented in order to optimize patients' diagnostic and therapeutic processes. We believe that this management strategy could offer a valuable opportunity for the optimization of preoperative and postoperative times, with a significant impact on patients' perceived quality of care, though amid a global healthcare emergency. Through the implementation of some key interventions, we managed to minimize healthcare personnel and patients' unnecessary risk of SARS-CoV-2 exposure, and promote a rational use of limited resources, while at the same time complying with general oncological principles.

Lean thinking strategies implemented during the COVID-19 pandemic need to be monitored carefully as they may lead to significant improvements in clinical practice, even beyond the current pandemic.

## Data Availability Statement

The raw data supporting the conclusions of this article will be made available by the authors, without undue reservation.

## Ethics Statement

Ethical review and approval was not required for the study on human participants in accordance with the local legislation and institutional requirements. The patients/participants provided their written informed consent to participate in this study.

## Author Contributions

FP, GDF, SM, and GP contributed in the conceptualization, methodology, and writing the original draft of the study. FP and GDF contributed in the formal analysis of the study. FP, GDF, SM, GD, EF, and GP contributed in the investigation, data curation, data collection, and revision and editing of the study. FP and GP contributed in the supervision and project administration of the study. All authors contributed to the article and approved the submitted version.

## Conflict of Interest

The authors declare that the research was conducted in the absence of any commercial or financial relationships that could be construed as a potential conflict of interest.
